# “Bonnet bypass” using a radial artery interposition graft—how I do it

**DOI:** 10.1007/s00701-025-06700-7

**Published:** 2025-10-31

**Authors:** Rajiv K. Khajuria, Milad Neyazi, Katharina Faust, Sajjad Muhammad

**Affiliations:** Department of Neurosurgery, Düsseldorf University Hospital, Heinrich-Heine University Düsseldorf, North Rhine-Westphalia, 40225 Düsseldorf, Germany

**Keywords:** Bonnet bypass, Extracranial-intracranial bypass, Revascularization, Common carotid artery occlusion, Superficial temporal artery, Radial artery interposition graft

## Abstract

**Background:**

Common carotid artery (CCA) occlusion frequently results in compromised hemodynamics of the ipsilateral hemisphere with risk of infarction but also a lack of blood flow in the ipsilateral superficial temporal artery (STA), requiring a more complex revascularization strategy than a standard extracranial-intracranial (EC-IC) bypass when indicated.

**Method:**

We describe the performance of a “Bonnet bypass” using a radial artery interposition graft (RAIG) from the contralateral STA to the ipsilateral middle cerebral artery (MCA). A groove is drilled in the skullcap to position the RAIG and reduce risk of mobilization and compression.

**Conclusion:**

The “Bonnet bypass” enables a revascularization procedure when the ipsilateral STA is not available as donor vessel.

**Supplementary Information:**

The online version contains supplementary material available at 10.1007/s00701-025-06700-7.

## Case introduction

Here, we illustrate our procedure in the scope of the case of a male patient, who at the age of 71 years presented with a sudden vision loss on the right eye due to central retinal artery occlusion. Further work-up revealed CCA occlusion on the right side with reduced cerebrovascular reserve capacity in a “Diamox” computed tomography (CT) scan, resulting in the indication for a bypass surgery. The patient did not experience any further neurological deficits as of now, one year following surgery and the bypass remained patent.

### Relevant surgical anatomy

CCA occlusion occurs rarely and often becomes clinically apparent in patients due to transient ischemic attacks (TIAs) or ischemic stroke and corresponding neurological symptoms [1, 4, 5]. Because of lack of blood flow in the ipsilateral STA, the affected patients are not amenable to the standard revascularization procedure in form of an EC-IC bypass [2]. Thus, more complex strategies are necessary to achieve revascularization, which can be accomplished by using a radial artery interposition graft from the contralateral STA to the ipsilateral MCA, usually in the M4-segment [3, 6].

### Description of the technique

#### Preparation of the RAIG

**Fig. 1:**
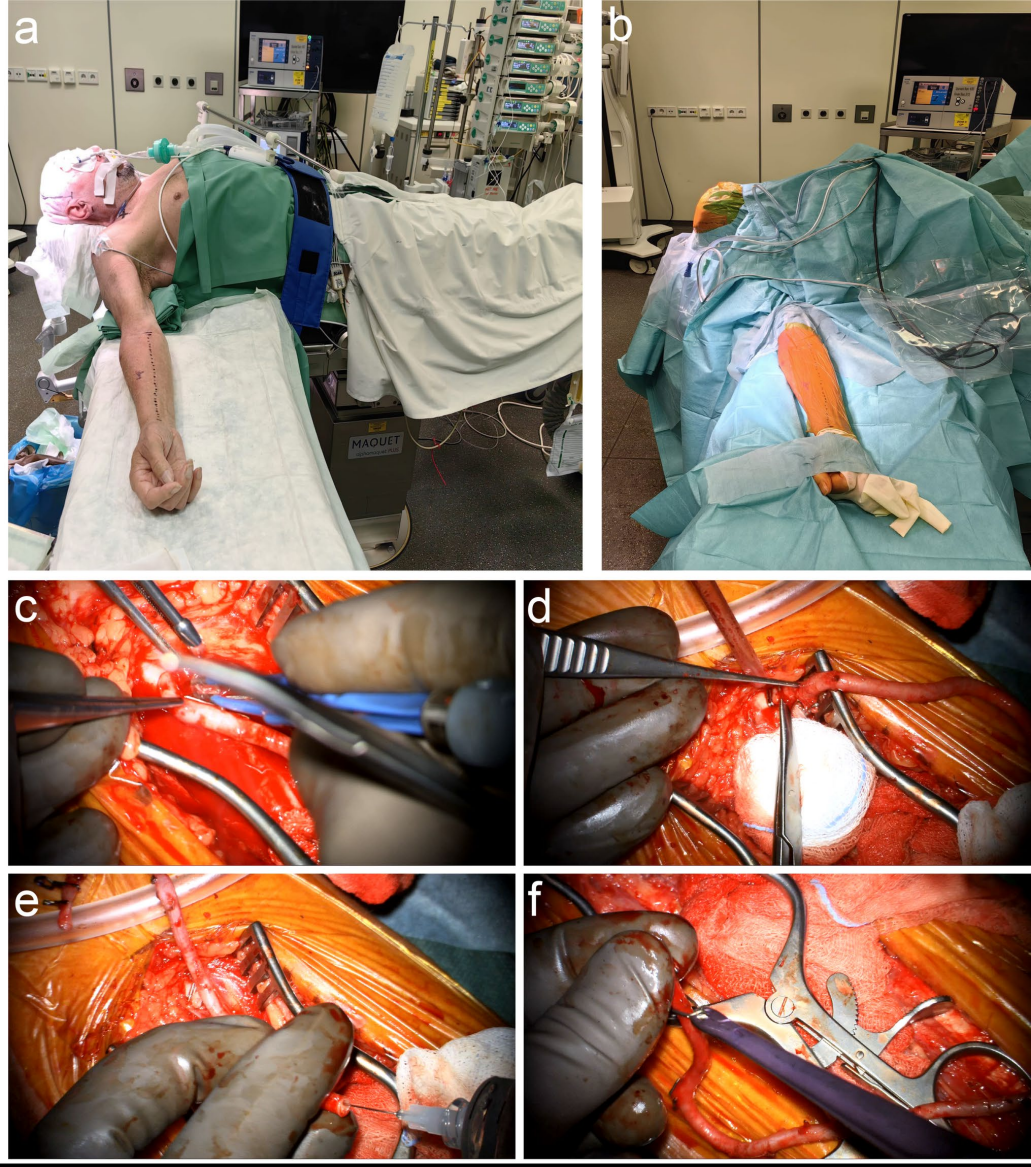
**a-b** The selected arm is placed on an arm mount and positioned laterally outwards. **c** The radial artery is dissected under the microscope using scissors, forceps and bipolar cauterization. **d** The in situ remaining ends are occluded by hemoclips and the graft is cut out with scissors. **e** The obtained radial graft is temporarily clip-occluded on one end and flushed with heparin and nimodipine for dilatation and to avoid vasospasm and thrombosis. **f** Then, also the other end is occluded until usage for anastomosis

Preoperatively, sufficient blood flow in at least one of the radial arteries is ensured by an Allen’s test and sonography. The selected arm is placed on an arm mount and positioned laterally outwards (Fig. 1a-b). An incision of approximately 20–23 cm is made according to the beforehand measured bicoronal length and the radial artery is dissected under the microscope using scissors, forceps and bipolar cauterization (Fig. 1c and Video 1). The in situ remaining ends are occluded by hemoclips, and the obtained radial graft is temporarily clip-occluded on one end and flushed with heparin and nimodipine for dilatation and to avoid vasospasm and thrombosis (Fig. 1d-e and Video 1). Then, also the other end is occluded until usage for anastomosis (Fig. 1f).

The RAIG can be obtained from either forearm depending on the preoperatively determined vascular status, but removal from the non-dominant side is preferred, when possible, to reduce potential health related restrictions in case of complications.

### Head positioning and preparation of the STA

**Fig. 2:**
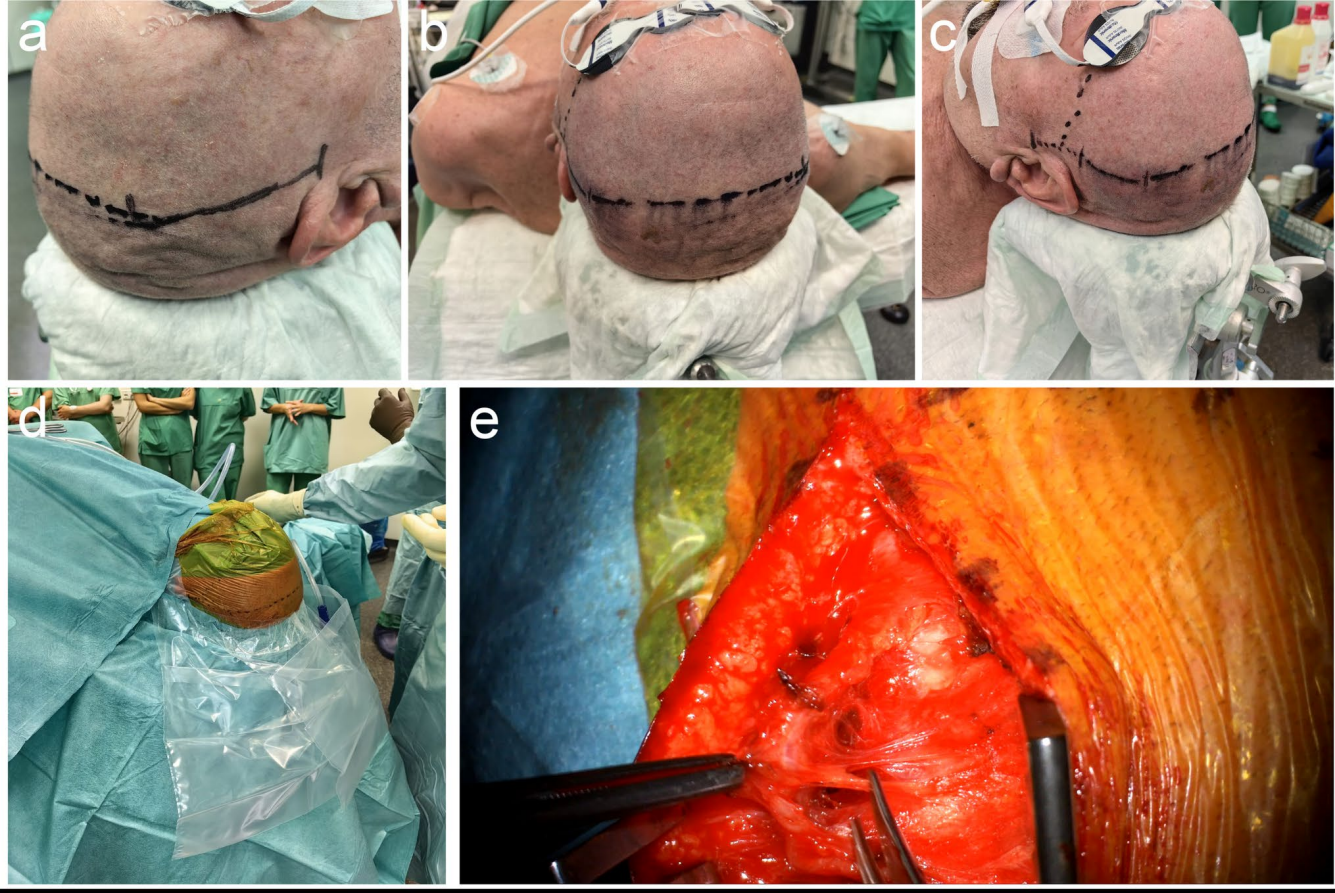
**a**-**d** The head is placed on a head mount without fixation in a Mayfield clamp to allow for mobilization during surgery. The planned incision and positioning of the RAIG over the convexity is marked. **e** The STA is dissected using scissors, forceps and bipolar cauterization

The head is placed on a head mount turning the head initially to the side of the CCA occlusion without fixation in a Mayfield clamp to allow for mobilization during surgery (Fig. 2a-d). The contralateral STA is identified by doppler ultrasound anterior of the tragus, usage of the parietal branch is preferred. A skin incision of 4 cm is made directly on top of the vessel, which is then carefully dissected using scissors, forceps and bipolar cauterization (Fig. 2e and Video 1). All steps for STA preparation are carried out under the microscope.

### Craniotomy and preparation of the MCA

**Fig. 3:**
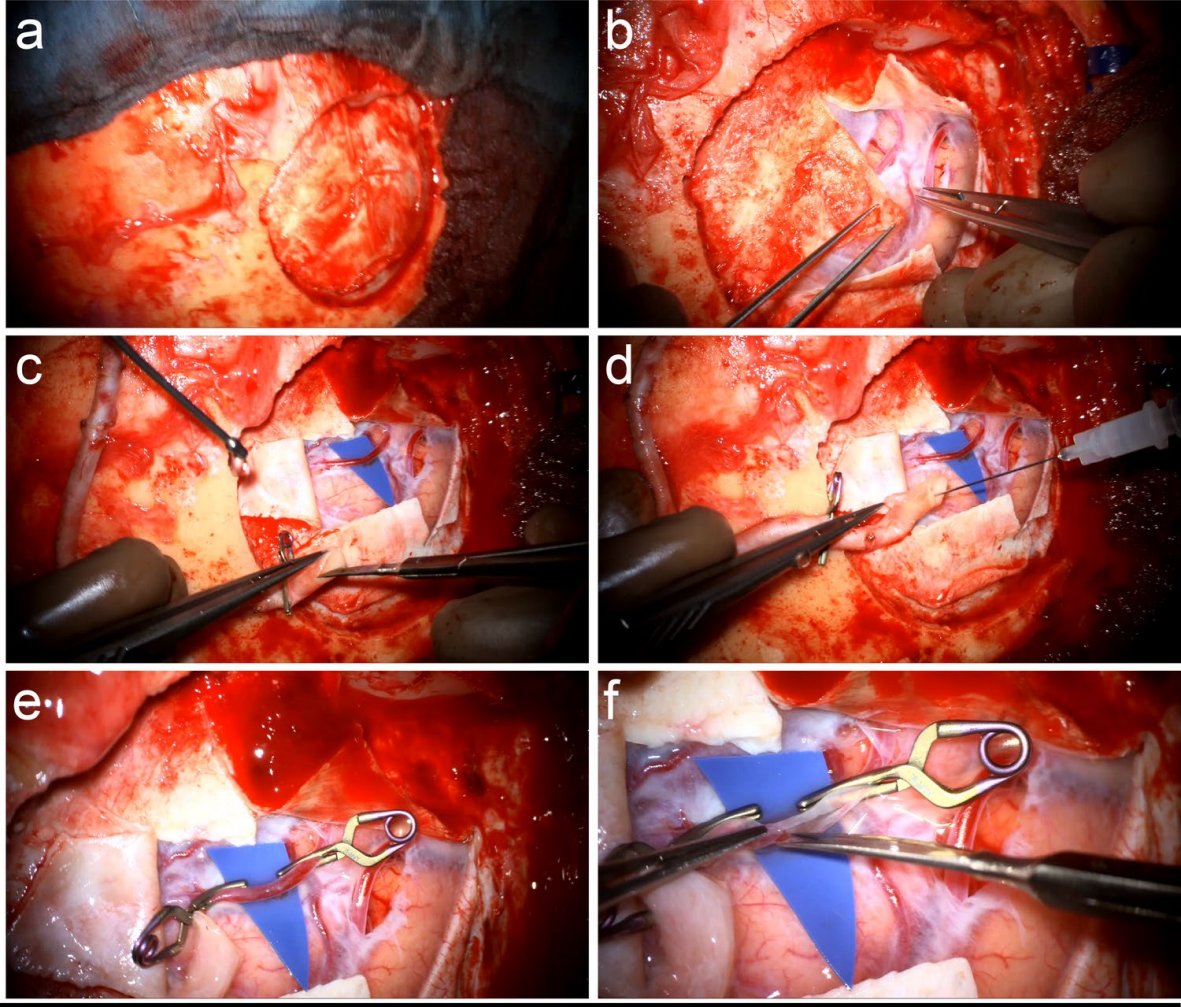
**a** A 4 x 4 cm craniotomy in the caudal area of the Sylvian fissure is performed and **b** the dura is opened in a Y-shaped manner. **c** The RAIG is prepared in fish-mouth technique for anastomosis and **d** once more flushed with heparin and nimodipine to prevent thrombosis and vasospasm. **e** The recipient M4-artery is temporarily clipped proximally and distally of the arteriotomy, **f** which is carried out with a microscalpel as well as microscissors and comparatively large to account for the size mismatch of the large RAIG and the small M4-artery

Following preparation of the STA, a bicoronal skin incision is performed for the planned placement trajectory of the RAIG, which is extended on the CCA occlusion side for the craniotomy, while the head is turned accordingly. A 4 × 4 cm craniotomy in the caudal area of the Sylvian fissure is performed and the dura is opened in a Y-shaped manner (Fig. 3a-b). A suitable M4-segment recipient artery is identified, and the radial artery interposition graft is prepared in fish-mouth technique for anastomosis (Fig. 3c and Video 1). Additionally, the RAIG is once more flushed with heparin and nimodipine to prevent thrombosis and vasospasm (Fig. 3d). The recipient vessel is temporarily clipped proximally and distally of the arteriotomy, which is carried out with a microscalpel as well as microscissors and comparatively large to account for the size mismatch of the large RAIG and the small M4-artery (Fig. 3e-f and Video 1).

### Anastomoses with the RAIG

**Fig. 4:**
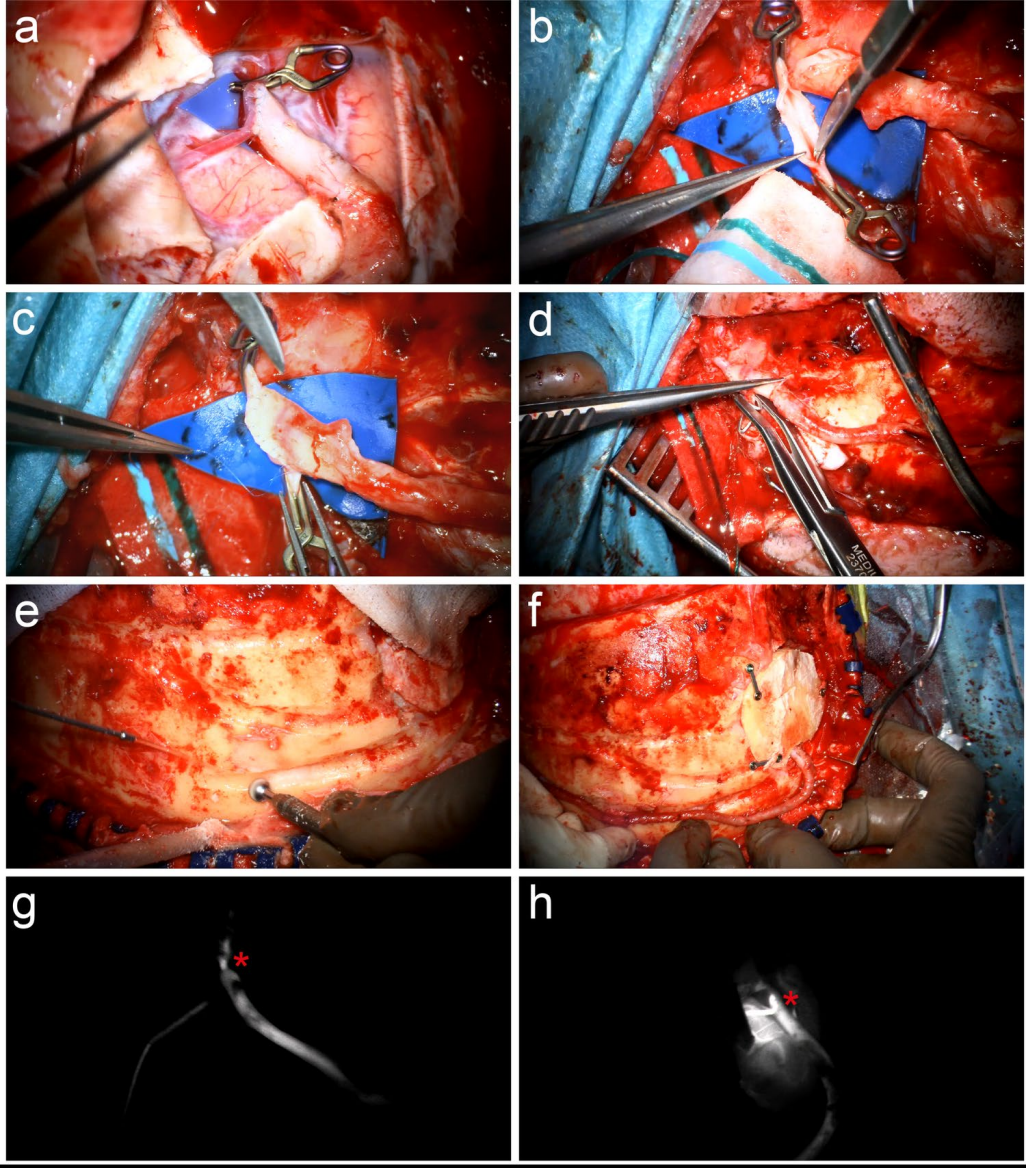
**a** First, the RAIG-M4 anastomosis is performed with simple interrupted sutures. **b** Then, a large arteriotomy on the STA is performed. **c** The STA-RAIG anastomosis is performed in an analogous manner. **d** A hemoclip is placed on the STA distally to the anastomosis to improve flow through the bypass. **e** A groove is drilled over the skull convexity **f** to place the RAIG and reduce its’ mobilization. ICG-VA confirms patency of **g** the STA-RAIG and **h** the RAIG-M4 anastomoses. The red asterisk in **g** and **h** is at the site of the anastomosis, respectively

First, the anastomosis between the RAIG end and side of the M4-segment artery is created using simple interrupted 10.0 Prolene sutures with a BV-2 needle (ETHICON) (Fig. 4a and Video 1). Then, following arteriotomy of the STA, the anastomosis between the other RAIG end and side of the contralateral STA is performed in an analogous manner (Fig. 4b-c and Video 1). The STA distally to the anastomosis is occluded with a hemoclip and may be cut (Fig. 4d and Video 1). These measures are not necessarily required but may improve flow through the bypass. A grove is drilled, in which the RAIG is positioned over the convexity to reduce its’ mobilization and potential compressing forces (Fig. 4e-f and Video 1). Flow through the anastomoses and the bypass graft is confirmed intraoperatively by indocyanine-green video-angiography (Fig. 4g-h and Video 1).

Postoperatively, systolic blood pressure is maintained between 120 and 160 mmHg for one day. A CT scan, including CT angiography and CT perfusion, is performed six hours following surgery to assess bypass patency and to evaluate for potential hypo- or hyper-perfusion syndromes.

**Fig. 5:**
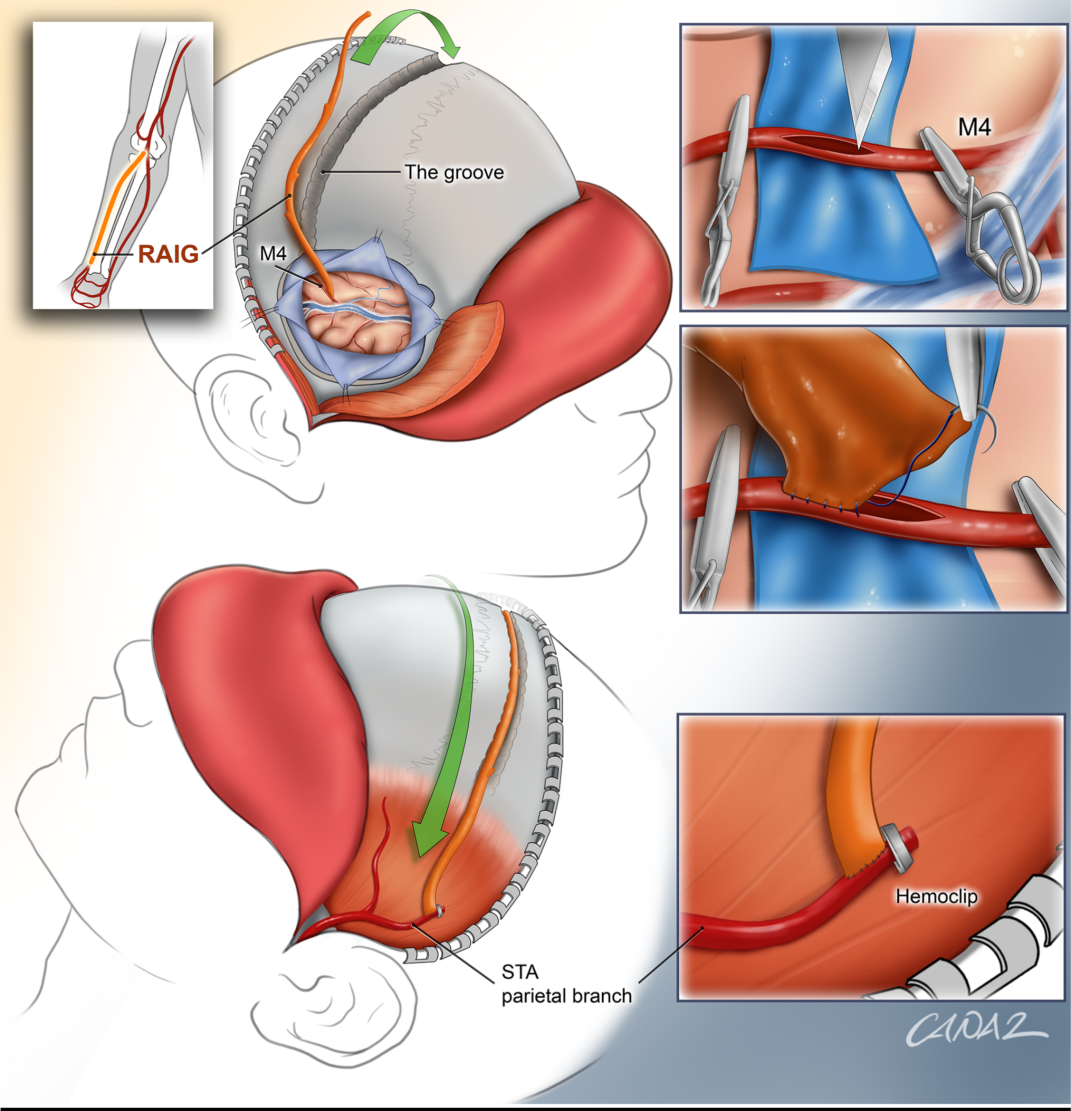
Shown is an illustrative overview of the Bonnet bypass using a RAIG. This includes the key steps of obtaining the RAIG (inlet on the top left), a craniotomy in the caudal area of the Sylvian fissure and a drilled groove over the skull convexity (upper head illustration), temporary trapping and a large M4-recipient artery arteriotomy (inlet on the top right) and the RAIG-M4 anastomosis (inlet in the middle right). The RAIG is placed in the drilled groove (lower head illustration) and the STA-RAIG anastomosis is performed (inlet in the bottom right). Optionally, a hemoclip may be placed on the STA distally to the anastomosis and the STA may be cut to improve flow through the bypass (inlet in the bottom right)

An illustrative overview of the key aspects of the whole procedure is shown in Fig. 5.

## Indications

The main indication is the necessity of a bypass in case of a CCA occlusion with a hemodynamically compromised ipsilateral hemisphere with recurrent transient ischemic attacks and high risk of infarction and a non-perfused STA on the ipsilateral side [1, 2, 4, 5]. But also other indications for bypass performance such as occlusion or high-grade stenosis of the internal carotid artery or trapping of an intracranial aneurysm may warrant a Bonnet bypass if the ipsilateral STA exhibits insufficient blood flow to qualify as donor vessel.

## Limitations

Performance of the Bonnet bypass as described requires the availability of a sufficiently long, well perfused radial artery interposition graft, which may be a limitation specifically in patients suffering from upper extremity artery occlusive disease, a population that may have a higher probability to also sustain CCA occlusion. This may be circumvented by the usage of an alternative graft such as a saphenous vein though [3, 6]. An increased risk of wound healing problems may occur due to poor formation of collaterals in the area of the ipsilateral STA.

A steno-occlusive condition also on the contralateral side may affect also the other STA, excluding the possibility of a Bonnet bypass.

The route of the RAIG over the convexity may make this revascularization construct somewhat vulnerable to forces impacting the head, which may restrict the patients in their potential activities to some extent.

Due to the scarcity of reports on Bonnet bypass cases, it is not clear if there may be higher risks for bypass failure or thrombosis overall as compared to more standard procedures, especially given the STA-RAIG size mismatch.

### How to avoid complications

Preoperative evaluation of the radial arteries by ultrasound and Allen-test as well as of the contralateral STA by ultrasound but also angiographic imaging are essential to ensure sufficient blood flow in these vessels for bypass performance. Implementation of all procedural steps under the microscope minimizes the risk of injury to these structures. For the first few months following surgery it may be advisable for the patient to wear a helmet during activities until the healing process is completed.

### Specific information for the patient

The Bonnet bypass offers a reasonable revascularization option for patients with an unusable STA on the ipsilateral side, requiring a more complex surgery than a standard EC-IC bypass though [3, 6]. The higher vulnerability for potential complications and related health restrictions appear acceptable considering the benefit of reducing the risk of infarction and thus, of potential neurological injury caused by CCA occlusion or other diseases requiring revascularization. The necessity of a life-long intake of platelet aggregation inhibitors exists often anyways in patients that are candidates for such a bypass.

## Keypoints

1. Preoperatively, sufficient blood flow in at least one of the radial arteries is ensured by an Allen’s test and sonography.
2. The head is placed on a head mount without fixation in a Mayfield clamp to allow for mobilization during surgery.
3. Following obtainment of the RAIG, first the STA is prepared on the side contralateral to the CCA occlusion, preferably the parietal branch.
4. The skin incision is extended bicoronally for the planned placement trajectory of the RAIG, which is extended on the CCA occlusion side for the craniotomy.
5. A 4 × 4 cm craniotomy in the caudal area of the Sylvian fissure is performed and the dura is opened in a Y-shaped manner
6. Comparatively large arteriotomies are performed on the M4-reciepient artery and the STA to account for the size mismatch to the RAIG.
7. The anastomoses are performed with simple interrupted sutures.
8. Occluding the STA distal to the anastomosis may improve flow through the bypass.
9. A groove is drilled in the skull convexity to place the RAIG and reduce its’ mobilization.
10. Patency of the anastomoses and the RAIG are confirmed by ICG-VA.

## Supplementary Information

Below is the link to the electronic supplementary material.

ESM 1 MP4 (945 MB)

## Data Availability

No datasets were generated or analysed during the current study.

## Abbreviations

CCA: Common carotid artery
STA: Superficial temporal artery
EC-IC: Extracranial-intracranial
MCA: Middle cerebral artery
RAIG: Radial artery interposition graft

## Declarations

This study complies with ethical guidelines, and the patient provided informed consent for the use of image and video material.
